# Inferential challenges when assessing racial/ethnic health disparities in environmental research

**DOI:** 10.1186/s12940-020-00689-5

**Published:** 2021-01-12

**Authors:** Tarik Benmarhnia, Anjum Hajat, Jay S. Kaufman

**Affiliations:** 1 Department of Family Medicine and Public Health & Scripps Institution of Oceanography University of California, San Diego, 9500 Gilman Drive, La Jolla, CA 92093 USA; 2 Department of Epidemiology, University of Washington, Seattle, WA USA; 3 Department of Epidemiology, Biostatistics, and Occupational Health, McGill University, Montreal, QC Canada

**Keywords:** Air pollution and health, Race/ethnicity, Causal inference, Social epidemiology

## Abstract

Numerous epidemiologic studies have documented environmental health disparities according to race/ethnicity (R/E) to inform targeted interventions aimed at reducing these disparities. Yet, the use of R/E under the potential outcomes framework implies numerous underlying assumptions for epidemiologic studies that are often not carefully considered in environmental health research. In this commentary, we describe the current state of thinking about the interpretation of R/E variables in etiologic studies. We then discuss how such variables are commonly used in environmental epidemiology. We observed three main uses for R/E: i) as a confounder, ii) as an effect measure modifier and iii) as the main exposure of interest either through descriptive analysis or under a causal framework. We identified some common methodological concerns in each case and provided some practical solutions. The use of R/E in observational studies requires particular cautions in terms of formal interpretation and this commentary aims at providing a practical resource for future studies assessing racial/ethnic health disparities in environmental research.

**Supplementary Information:**

The online version contains supplementary material available at 10.1186/s12940-020-00689-5.

## Introduction

Over the last decade, there has been increasing interest in assessing the role of environmental determinants in health disparities [1–3]. Among the social factors that can influence the distribution of hazards or modify the impact of environmental factors on health, race/ethnicity (R/E) has been among the more commonly studied [4, 5]. Numerous epidemiologic studies have been conducted to document environmental health disparities according to R/E to inform targeted interventions toward reducing these disparities [6–8]. The classification of race/ethnic groups used in public health research captures long-established and systemic consequences of political, historical, and economic structures and social constructs [9–11]. Race/ethnicity in epidemiological studies may operate through various pathways such as differential treatment, social isolation and structural racism to generate observed disparities [12, 13].

Nevertheless, the use of R/E implies numerous underlying considerations for epidemiologic studies that are often not carefully considered. In parallel, various methodological challenges have recently been highlighted in the social epidemiology literature when using R/E as a variable in statistical models [14, 15] and such challenges have not been discussed explicitly in the context of environmental research.

In this commentary, we first briefly provide a historical overview of how R/E has been used in epidemiological research and discuss interpretation and inferential challenges. We then introduce the potential outcomes (counterfactual) framework and related assumptions for identification in causal inference. Finally, we assess how R/E is used in environmental studies, using air pollution as a case study, highlight some frequent methodological challenges that relate to the role of environmental determinants in health disparities and propose solutions for further studies that consider racial/ethnic disparities in health.

## The use of R/E as a social construct in epidemiological research: a brief historical overview

Every human civilization has crafted categories of race, ethnicity, or caste as a means of creating a hierarchy among groups within the population on the basis of appearance, lineage or geographic origin. These categories are distinct from groupings based on religion or ideology in being fixed at birth, although this distinction can be blurred, as in the case of those who identify as Jewish. The definitions of and boundaries between groups are generally highly mobile over time and place, often as the result of political mobilization and popular agency [16, 17]. Because race and ethnicity have deep resonance within human societies for family formation (partnering, marriage and adoption) and access to resources such as employment, housing and services, they are also highly predictive of structural inequalities and the distribution of health and disease [18].

Racial and ethnic variables have therefore been thoroughly integrated into epidemiological and public health research all over the world, and especially so in countries that created stark racial hierarchies through race-based slavery (e.g. USA, Brazil), racial apartheid (e.g. South Africa), colonization (e.g. Rwanda) or the systematic disenfranchisement of a native population by European settlers (e.g. Canada, Australia, New Zealand). In all of these settings, R/E becomes a major axis of epidemiologic risk, along with sex/gender and social class/position [19, 20]. Policies differ by country, but in the US, the centrality of the R/E experience in patterning exposures, risks and access to services motivates the government to collect these variables on official censuses, health surveys and other administratively gathered databases [21]. These data are then used in surveillance, to detect disparities, and in documenting evidence of racial discrimination in access to resources and services [22].

Epidemiology and clinical medicine have exploited the availability of R/E information in datasets, especially in the US, to generate a vast literature focused on these categories. Even when R/E is not the focus of the research, it is rare to find a US biomedical article on human subjects that does not refer to R/E in the description of the study population or use this information as a control variable in analyses. This has led to numerous recommendations, critiques and lamentations about the ambiguity, confusion or misinterpretation that such ubiquitous and reflexive use has engendered [9].

## Causal interpretation(s) of race/ethnicity in epidemiology

A first challenge when using racial or ethnic categories to define health disparities is related to the ambiguous interpretation or active misinterpretation of the” effects” of R/E. Race and ethnicity as epidemiologic variables have been notably theorized through the counterfactual (or potential outcomes) framework [23] which is widely used in contemporary epidemiological research as well as other fields.

### Introducing the potential outcomes framework

The potential outcomes (PO), or counterfactual framework is widely considered as fundamental to understanding causal effects in epidemiology [24, 25]. For example, if one is interested in estimating the effect of a heat wave event on stroke rates, it requires contrasting the observed rate with an estimate of the unobserved stroke rates in the same world on the same day if the heat wave had not occurred. In this framework, causal effects are often defined as an average difference (or ratio) between two potential outcomes, one observed (factual) and the other unobserved (counterfactual), although in many settings, both halves of the contrast are counterfactual [26]. The *fundamental problem* of causal inference in this context is that, by definition, at least one half of the contrast is necessarily impossible to observe [27]. Therefore, identification strategies and related assumptions have been formulated when estimating causal effects [28].

Three main assumptions are usually formulated when aiming to identify causal effects under the potential outcomes framework: exchangeability, positivity and consistency. Exchangeability means that the counterfactual outcome and the actual treatment are independent. Some authors also refer to unconfoundedness of the assignment to exposure [24]. Practically, this refers to the absence of (measured or unmeasured) common causes of the exposure and outcome. Randomization is notably expected to achieve exchangeability but different identification strategies can be used for observational research including instrumental variables or difference-in-differences, for example, to achieve exchangeability [29, 30]. Exchangeability can also be addressed by achieving covariate balance between exposure groups for measured confounders; different analytic or design strategies can be used in this regard, such as standardization and matching. Although intuitively straightforward for most exposures, this appeal to randomization as a mechanism to achieve covariate balance is inconceivable for R/E variables, since R/E cannot by assigned in a trial, except in artificial circumstances [13]. Moreover, the common causes of R/E variables are generally diffuse historical and political processes that are beyond the scope of any dataset.

Positivity, the next assumption of the potential outcomes framework, requires that individuals in every stratum of the covariates have a non-zero probability of being in the exposed group and of being in the unexposed group. This assumption can be violated when studying R/E health disparities. For example, Messer et al. [31] showed that, in the case of the effects of SES and racial residential segregation on preterm birth, positivity violations may lead to meaningless conclusions because effect estimates from regression modeling were based on little or no actual data. This has been described as “structural confounding” in the sense that certain combinations of R/E and social status are rare in the population as a function of structured relations between social groups. Messer et al. noted that areas of sparse or missing data in their analyses were a structural reality about the systematic co-occurrence of racial segregation and poverty, a relationship that was mandated by law until the Civil Rights Act of 1965.

Finally, the consistency assumption requires that all exposed individuals receive the same version of treatment. This is also sometimes referred to as the stable unit treatment value assumption (SUTVA) that additionally requires no interference between treated and control groups [32]. This assumption implies that the exposure of interest must be defined with sufficient precision that any variation within the exposure specification would not result in a different outcome [33]. It has been shown that this assumption is commonly violated in social epidemiological research when using variables such as education or income [34], but also when using race/ethnicity as an exposure of interest. Indeed, it is possible to consider two distinct situations where the main exposure of interest is: i) R/E, ii) another variable on the pathway between R/E and the health outcome of interest (e.g. residential exposure to fine particulate matter, noise, greenspace) [9]. Considering R/E as the exposure of interest can be thus seen as ambiguous and typically violates the consistency assumption under the potential outcomes framework.

### Manipulability of the exposure and different perspectives about the use of R/E in epidemiology

Another identified challenge when using R/E as the exposure of interest for inferential research (in contrast to descriptive and predictive research) is related to the potential manipulability of the exposure. Indeed, many authors have argued that R/E does not meet the criteria for a well-defined intervention, implying that the required causal assumptions discussed above may not hold [23, 35, 36]. Yet estimating the effect of R/E seems more conceivable in some contexts if R/E represents the experience of individual discrimination or structural inequality. For example, self-reported race for job applications [37] or randomizing fictitious subjects to be classified by race and observe differential diagnosis or treatment by a clinician [38] have been described as well-defined and potentially manipulable interventions. Even so, some authors [39] point out that some ambiguity remains regarding the meaning of discrimination.

Various authors [40, 41] have proposed that observational studies should specify an ideal RCTs as a target for the causal effect to be estimated, and implicitly suggest that any non-manipulable exposure is not eligible as an exposure of interest. In parallel, other authors propose a non-interventionist interpretation to such causal reasoning allowing for the consideration of R/E as an exposure of interest and reject the argument that the causal effects of race require any hypothetical manipulation whatsoever [42–44].

In this context, VanderWeele and Robinson [15] proposed two possible interpretations of the “effects” of race, as the main exposure of interest. First the authors proposed a so called “stronger” interpretation of race where “once the components of race are specified, the effect of race corresponds to the joint effects of these specific components for which interventions are at least somewhat more conceivable”. Secondly, they proposed a “weaker” interpretation where “R/E regression coefficients in a model with certain control variables are interpreted as estimating what would happen to an observed health inequality if certain socioeconomic status distributions were set to something other than what they in fact were”. They suggest estimating direct and mediated inequality measures by including in the same model for the health outcome a R/E variable contrast, such as Blacks versus Whites, a “mediating” variable of interest, such as residential exposure to fine particulate matter, and potentially any number of mediator-outcome confounders such as age. From this model, the coefficient for the R/E variable can be interpreted, for example, as the inequality in the health outcome remaining had the distribution of residential fine particular matter (PM2.5) for the Black population been set to the PM2.5 exposure of the White population with the same values of the confounders. They also proposed to estimate the magnitude of the “mediated inequality effect” through residential PM2.5 by comparing the inequality measure before/after controlling for PM2.5 and mediator-outcome confounders. However, traditional regression adjustments would lead to biased estimates in the presence of another mediator between R/E and Y that itself affects PM2.5 [45] and in that case alternative analytical strategies including inverse probability weighting (IPW), G-computation or stochastic mediation analysis would be required. Following VanderWeele and Robinson [15], Jackson and VanderWeele [46] proposed a comprehensive set of hypothetical interventions under the potential outcome framework to quantify impacts on social disparities of interest. The authors stressed how important it is to specify the hypothetical intervention scenario and related implications for health disparities and how each scenario corresponds to distinct analytical strategies.

Besides the potential outcome framework, it is worth mentioning other frameworks to study R/E disparities in health, such as the theory of fundamental causes [47]. An interesting facet of this framework is the distinction between replaceable mechanisms, which are framed as not directly actionable, and flexible resources that need to be targetted to ultimately reduce R/E inequalities in health. Naimi [48] proposed to integrate the PO framework and fundamental cause theory to generate evidence on the potential interventions to mitigate racial/ethnic disparities in health. He noted that “[…] contrasts of the risk of a health outcome between racial/ethnic groups can validly be interpreted as quantitative expressions of long-standing race relations and are thus not without meaning.” He also argued that racial/ethnic disparity questions “are counterfactual in that they relate to the risk of a health outcome among different racial or social groups that would be observed if a third variable was modified.” Similar interpretations have been suggested by Krieger [49].

This ongoing vigorous discussion shows that R/E variables can be approached in different ways when assessing health disparities. One must therefore pay particular attention to the interpretation of this variable, be transparent about the intended interpretation and check that assumptions required for causal inference are sufficiently satisfied and appropriate identification strategies are mobilized. Whatever causal framework is used to highlight potential social disparities in health, the use of race/ethnic variables requires particular caution in terms of its formal interpretation when included in statistical models. This contrasts with the more cavalier practice often encountered in the epidemiologic and biomedical literature.

## How race/ethnicity variables are used in environmental epidemiology

Although discussions about the interpretation of R/E may be relatively common in the social epidemiology literature, they are largely absent in environmental epidemiology. Here, we provide examples of environmental health studies that included R/E indicators in order to describe the different ways it has been used in these studies. Using Google Scholar, we selected a convenience sample of 15 illustrative studies related to air pollution and health in North America from 2014 to 2019, as this is an active area of research where health disparities related to air pollution are a great concern. Details about keywords are provided in Table 1. We focus on how R/E variables are considered in the US context but it is worth mentioning that potential mechanisms linking R/E and outcomes vary among countries. Furthermore, some of the issues we highlight in this paper can be applicable to other SES variables including education and income. It is important to acknowledge that, as R/E is largely fixed at birth, all hypothetical interventions are on intermediates, and it is possible to improve education or income with policy interventions [65].

**Table 1:** Illustrative papers that used race/ethnicity in relation to air pollution exposure or effects on health

Study	Aims	Outcome	Air pollutants	How race/ethnicity variables were used
**R/E as a confounder**				
Nobles et al., 2019 [50]	Impact of air pollution on fetal growth restriction	Physician diagnosed fetal growth restriction	SO2, O3, NOX, NO2, CO, PM10, PM2.5	As a confounder, maternal R/E was included in models with maternal age, race/ethnicity, pre-pregnancy body mass index, smoking, alcohol, parity, insurance, marital status, asthma and temperature.
McGuinn et al., 2019 [51]	Impact of air pollution on cardiovascular disease risk	Lipoprotein levels	PM2.5	As a confounder, R/E was included in models with age, sex, history of smoking, area-level education, urban/rural status, body mass index, and diabetes.
Bragg-Gresham et al., 2018 [52]	Impact of air pollution on the prevalence of diagnosed chronic kidney disease in US medicare population	Chronic kidney disease	PM2.5 county level	As a confounder, R/E was included in models with age, sex, hypertension, diabetes, and urban/rural status.
Ng et al., 2017 [53]	Impact of air pollution on birth weight	Term low birth weight	PM2.5	As a confounder, maternal R/E was included in models with maternal age, maternal education, gestational age, year of birth, gestational apparent temperature exposure, and percentage of households below poverty line at the ZCTA level Also as an effect measure modifier (multiplicative scale)
Gray et al. 2014 [54]	Impact of air pollution and SES variables on birth outcomes	Low Birth Weight, Preterm Birth	O3 and PM2.5	As a confounder; R/E was included in models with maternal education, maternalage at delivery, and census tract-level median household income
Chen et al. 2015 [55]	Impact of air pollution on brain volumes in older women	Cognitive decline (measures of gray matter and normal appearing white matter)	PM2.5	As a confounder; analysis used a staged modelling approach where minimally adjusted models were adjusted for R/E and other covariates (not SES) and more fully adjusted models included both R/E and SES (education, family income, and employment status) and other covariates. Additional analyses restricted to non-Hispanic White women.
**R/E as a EMM**				
Leiser et al., 2019 [56]	Effects of air pollution on spontaneous pregnancy loss	Spontaneous pregnancy loss	PM2.5, NO2, O3	As an effect measure modifier (multiplicative scale, in a case crossover design)
Laurent et al., 2016 [57]	Impact of air pollution on birth outcomes	Low birth weight	PM, NO2, O3	As an effect measure modifier (multiplicative scale)
Delfino et al. 2014 [58]	Impact of air pollution on asthma and R/E as a vulnerability factor	Asthma-related hospital morbidity	Traffic-related air pollution	As an effect measure modifier (multiplicative scale, in a case crossover design)
Strickland et al. 2014 [59]	Impact of air pollution on children’s asthma and R/E as a vulnerability factor	Emergency department for asthma or wheeze among children 2 to 16 years of age	CO, NO2, PM2.5, O3	As an effect measure modifier (multiplicative scale). Heterogeneity tests were conducted.
**R/E as a main exposure**				
Grineski & Collins, 2018 [60]	Disparities in exposure to neurotoxicants in US public schools	Air pollution	neurotoxicants from US Environmental Protection Agency’s National Air Toxics Assessment (NATA).	As the main exposure of interest, adjusting for school district effects.
Tonne et al., 2018 [61]	Inequalities in air pollution exposure by socio-economic status and racial/ethnic groups	Air pollution	PM2.5, NO2	As the main exposure of interest, adjusting for age, age squared, ethnicity, household income, area-level income deprivation, and a random effect for household.
Kravitz-Wirtz et al. 2016 [62]	Inequalities in air pollution exposure by racial/ethnic groups	Air pollution	NO2, PM2.5, and PM10	As the main exposure of interest, adjusting for age, family size, income, employment, housing tenure, metropolitan level segregation and industrial share
Jones et al. 2014 [63]	Inequalities in air pollution exposure by racial/ethnic group and racial residential segregation	Air pollution	PM2.5 and NOx	As the main exposure of interest, adjusting for education, annual family income, and neighborhood median family income
Jones et al. 2015 [64]	Decomposition of the total effect between R/E and intima-media thickness using air pollution exposure as a mediator.	Intima-media thickness	PM2.5 and NOx	As the main exposure of interest, adjusting for age, education, annual family income, smoking status, pack-years of smoking, BMI, diabetes status, systolic blood pressure, total and HDL cholesterol, antihypertensive medication use and statin use.

The keywords used for the literature review were: (race OR ethnic* OR black OR African American OR Hispanic OR Latino OR minorities) AND (Air pollut* OR air quality OR urban pollut* OR ambient air pollution OR atmospheric pollut* OR air contamination OR ambient particulate matter” OR air pollution control OR air-pollution)

For each type of application, we aimed to identify some persistent concerns and propose solutions for future studies. This overview does not constitute an assessment of the overall scientific value of the cited papers; it is only a review of the methodology employed for the different uses of R/E variables. Table 1 presents an overview of illustrative papers that used R/E in relation to air pollution exposure or effects on health. It is important to note that a formal interpretation of the “effects” of race was rarely provided in the selected papers. Thus, we recorded the ways that R/E variables were used based on our best interpretations of the text provided in each article.

### Race/ethnicity as a confounder

Race/Ethnicity is often introduced in regression models as a covariate representing a potential confounder in the relationship between air pollution and studied health outcomes [50–55]. This is by far the most common way that R/E has been incorporated in air pollution health effects studies.

In the US context, R/E is a powerful predictor of SES, as it shapes one’s access to education, occupational opportunity, ability to accumulate wealth and where someone lives. Yet, when air pollution is the exposure, using individual R/E can be seen as a proxy for racial residential segregation and not a direct cause of individuals’ air pollution exposure. Such approximation should be clearly motivated and stated when using individual R/E as a confounder when air pollution is the exposure of interest. Furthermore, neighborhood-level indicators of racial segregation [66] seem to be more strongly associated with air pollution then individual level traits using socio-economic indicators for example [67].

Another situation sometimes encountered is the reporting, and more problematically, the interpretation of regression coefficients for R/E from a model where the exposure of interest is air pollution, as exemplified in a paper that assessed the impacts of race, social factors and air pollution on birth outcomes [54]. In such settings, air pollution is considered as a mediator on the pathway between R/E and birth outcomes and the interpretation of the R/E coefficient should be viewed with caution (as discussed above). Such practices [68] have been critiqued as a source of potential confusion in many areas of epidemiologic research. This problem can be easily prevented by only presenting and interpreting coefficients related to the main exposure of interest (e.g. PM2.5). For example, we can consider individual-level exposure to PM2.5 as our main exposure of interest, serum C-reactive protein (CRP) levels as the outcome of interest and neighborhood R/E composition as the only confounder (for simplicity). In this setting, we can simply use a multivariable linear regression model where CRP is the dependent variable and PM2.5 and neighborhood R/E are included as independent variables and get a coefficient (i.e. slope) for each of these two independent variables. The coefficient for PM2.5 will approximate our causal quantity of interest, namely the change in CRP due to PM2.5 conditioned on the distribution of R/E. But the interpretation of the coefficients for R/E is quite different. Indeed, in this context PM2.5 is considered as an intermediate between R/E and CRP and this R/E coefficient represents the association between R/E and CRP removing the pathway through PM2.5. This is the reason for which we recommend to only present and interpret coefficients related to the main exposure of interest for such inferential research questions. Yet, it is possible to consider an environmental exposure (e.g. PM2.5) as a mediator between R/E and a given outcome and we discuss such setting in a subsequent section below.

In parallel, as discussed above, the positivity assumption can be particularly relevant in environmental epidemiology studies and requires careful attention. For example, considering exposure to specific hazardous industrial emissions such as polycyclic aromatic hydrocarbons (PAHs), exposed individuals may be a very distinct population from all potential unexposed individuals or it is possible that exposure to such industrial emissions and poverty systematically co-occur. In this context, it is notably important to ensure that there is covariate balance between exposed and unexposed individuals so that unexposed individuals are comparable in regards to measured confounders including R/E. Propensity score methods [69], for example, can assist in checking covariate balance and selecting the appropriate control group and thereby improve inference of the effects of air pollution on a given health outcome.

### Race/ethnicity as an effect measure modifier

It is first important to clarify how the concepts of effect measure modification (EMM) and interaction differ under the PO framework. Considering an exposure E, an outcome Y and a third variable Z, the concept of EMM (where the effect of E on Y is modified by Z) refers to the causal effect of E on Y only and not to the causal effect of Z. This means that only E is considered to be a variable on which we hypothetically intervene and for which exchangeability, positivity and consistency apply. In parallel, the concept of interaction refers to a joint causal effect of two distinct treatments E and Z. Identifying interaction thus requires exchangeability, positivity, and consistency for both E and Z [70].

That being said and following the non-manipulability feature of R/E variables described above, we can assume that R/E variables may be included as potential effect measure modifiers in order to represent some differential vulnerability or susceptibility by R/E status of the air pollution impacts on health [57–59]. To do this, studies typically included R/E in their regression models as product terms with exposure to air pollutants. EMM can occur on both absolute and multiplicative scales. In the papers we reviewed, it is notable that these product terms were most often included in multiplicative models (e.g. logistic, Poisson) and by default represent deviations from multiplicative joint effects and not deviations from additivity. Many papers discuss why the absolute scale is a more appropriate scale for inferring public health policy implications [71]. For authors interested in quantifying EMM on the additive scale from multiplicative models, some tools have been proposed to facilitate this calculation. The Relative Excess Risk due to Interaction (RERI) or Interaction Contrast Ratio (ICR) [72] as well as the synergy index (SI) or ratio of joint exposures (RJE) have all been proposed to measure additive interaction based on relative measures such as risk ratios or odds ratios [73]. Knol and VanderWeele [73] recommend to assess and report effect modification on both absolute and relative scales.

Other studies conducted stratified analyses to assess potential vulnerability to air pollution health effects [56, 58]. While not directly observed in the selected papers, an error that is nevertheless common in observational studies is that a “significant” exposure effect in one stratum is considered distinct from a “non-significant” exposure effect in another stratum [74]. As Gelman & Stern describe this error, the “difference between significant and not significant is not itself statistically significant”. Therefore, if statistical testing is used to show some evidence of an association between air pollution and a given health outcome among one R/E group, but this relation is not observed to be “significant” in another R/E group, it does not necessarily imply that the effect of air pollution is heterogeneous across the two R/E groups. Some simple solutions exist to deal with these issues. For example, if analyses are conducted across different R/E strata, conducting heterogeneity tests such as the Cochran Q test can provide statistical confirmation of EMM [75]. These heterogeneity tests can be used on both additive and multiplicative scales, although may be underpowered [76]. It is also possible to directly test the ratio or the difference between stratified effect measures [77]. In environmental epidemiology, some examples of using a ratio of risk ratios have been recently published [78, 79]. Finally, if a time series analysis is used, which is common in studies investigating acute health impacts of air pollution, it is also possible to directly address the association between air pollution and the intra-population disparities using daily differences between 2 or more groups [80]. Note that the more general issues related to null hypothesis significance testing are not discussed here, but we include in supplemental material a list of papers that discuss such issues and provide solutions.

### Race/ethnicity as the main exposure of interest

Finally, some studies have considered R/E as the main exposure of interest and air pollution as the outcome of interest either through descriptive analysis or under a causal framework. Studies that adjust on other variables thought to be confounders, such as age and sex, are implicitly operating under a causal framework, and therefore invoking the challenges discussed in previous sections. This is because confounding is itself a causal construct. In the papers reviewed, two specifications can be observed in this regard.

In the first specification, no health outcome is included; instead understanding inequities in air pollution exposures is the objective as with many environmental justice studies [60–64]. The inclusion of SES variables which may be on the causal pathway between R/E and air pollution may result in some attenuation of the association of interest. It should be noted, however, that the underlying causal mechanism that creates the association between R/E and locally undesirable land uses (LULU) in environmental justice studies has been widely debated and two non-exclusive mechanisms have been proposed. Some evidence indicates that minority communities are targeted for the placement of polluting facilities (disproportionate siting: disadvantaged R/E precedes LULU) [81–83]; while other research suggests that demographic changes occur after a hazardous facility has been placed in an area (post-siting demographic change: LULU precedes disadvantaged R/E) [84, 85].

In the second, far less common specification, a health outcome is included and the question of interest is to formally decompose the total effect between R/E and a given health outcome into an indirect effect through exposure to air pollution and a direct effect representing the effect of R/E through other pathways [64]. Treating air pollution as a mediator is potentially supported by the environmental justice theory of disproportionate siting. In this approach, a variety of different analytic approaches can be applied.

The use of causal mediation analyses has expanded in social epidemiology [86] over the last decade, and more recently in environmental epidemiology studies examining a variety of environmental exposures [87–90]. Besides the identification challenges related to the non-manipulability and consistency violation of R/E variables that are described above, some additional assumptions are required to estimate causal effects when conducting mediation analyses [91], including the absence of unmeasured confounders of the exposure-outcome, mediator-outcome and exposure-mediator associations (only required when estimating natural effects), as well as the absence of mediator-outcome confounders which are also affected by the exposure. As previously emphasized, if the decomposition of R/E disparities according to one or more environmental pathways is of interest, we strongly recommend to clearly specify the hypothetical health disparities scenario that is targeted and adopt an appropriate estimation strategy.

In parallel, econometric methods such as the Oaxaca-Blinder decomposition [92–94], as well as decomposition of inequality metrics, such as the concentration index [93, 95, 96], enable the simultaneous estimation of the contribution of multiple environmental exposures, to R/E disparities in a given health outcome under policy-relevant counterfactual intervention scenarios [97]. Jackson and VanderWeele [46] show that under some circumstances, Oaxaca-Blinder decomposition and mediation analysis coincide. They also explain that when there is time-dependent confounding, the Oaxaca–Blinder technique would lead to selection bias and should not be used. In this context, mediation analysis can be preferable with appropriate methods to deal with time-varying confounders, such as marginal structural models and G-computation. Furthermore, in a recent paper, the same authors emphasize the importance of clarifying the interpretation of variables representing a social construct when applying decomposition techniques in order to provide interpretable and actionable estimates for addressing intersectional health disparities [98].

## Conclusions

In this commentary, we described the current state of thinking about interpreting R/E variables in epidemiologic studies and provided examples of how R/E is typically used in environmental epidemiology. Although there are ongoing debates about how best to use and interpret R/E in observational studies more broadly, at the very least it is clear that authors should state unambiguously how they are conceptualizing R/E in a given study, provide thoughtful interpretation of R/E variables and evaluate causal inference assumptions as they relate to R/E. We identified three ways that R/E variables are used, highlighted some frequent methodological concerns and proposed solutions for further studies when assessing racial/ethnic health disparities in environmental research.

## Supplementary Information

**Additional file 1.**

## Data Availability

Not Applicable
