# Supplementary material for: Inferential challenges when assessing racial/ethnic health disparities in environmental research
Source: Environ Health. 2021 Jan 12;20:7. doi: 10.1186/s12940-020-00689-5 (PMC7802337; doi:10.1186/s12940-020-00689-5)
Supplement: Supplementary file 1 — Additional file 1. [file 12940_2020_689_MOESM1_ESM.docx]

**Supplemental Material**

**Title: Inferential challenges when** **assessing racial/ethnic health disparities in environmental research**

**Recommended papers null hypothesis significance testing in epidemiology**

- Wasserstein, R. L., & Lazar, N. A. (2016). The ASA’s statement on p-values: context, process, and purpose. The American Statistician, 70(2), 129-133.
- Amrhein, V., Greenland, S., & McShane, B. (2019). Scientists rise up against statistical significance.
- Greenland, S., Senn, S. J., Rothman, K. J., Carlin, J. B., Poole, C., Goodman, S. N., & Altman, D. G. (2016). Statistical tests, P values, confidence intervals, and power: a guide to misinterpretations. European journal of epidemiology, 31(4), 337-350
- Lash, T. L. (2017). The harm done to reproducibility by the culture of null hypothesis significance testing. American journal of epidemiology, 186(6), 627-635.
